# Prototype Gastro-Resistant Soft Gelatin Films and Capsules—Imaging and Performance In Vitro

**DOI:** 10.3390/ma13071771

**Published:** 2020-04-09

**Authors:** Bartosz Maciejewski, Vishnu Arumughan, Anette Larsson, Małgorzata Sznitowska

**Affiliations:** 1 Department of Pharmaceutical Technology, Medical University of Gdansk, 80-416 Gdansk, Poland; bartosz.maciejewski@gumed.edu.pl; 2 Department of Chemistry and Chemical Engineering, Chalmers University of Technology, 412 96 Gothenburg, Sweden; vishnu.arumughan@chalmers.se (V.A.); anette.larsson@chalmers.se (A.L.)

**Keywords:** gelatin, gastro-resistant, films, capsules, structure, drug release

## Abstract

The following study is a continuation of the previous work on preparation of gastro-resistant films by incorporation of cellulose acetate phthalate (CAP) into the soft gelatin film. An extended investigation on the previously described binary Gelatin-CAP and ternary Gelatin-CAP-carrageenan polymer films was performed. The results suggest that the critical feature behind formation of the acid-resistant films is a spinodal decomposition in the film-forming mixture. In the obtained films, upon submersion in an acidic medium, gelatin swells and dissolves, exposing a CAP-based acid-insoluble skeleton, partially coated by a residue of other ingredients. The dissolution-hindering effect appears to be stronger when iota-carrageenan is added to the film-forming mixture. The drug release study performed in enhancer cells confirmed that diclofenac sodium is not released in the acidic medium, however, at pH 6.8 the drug release occurs. The capsules prepared with a simple lab-scale process appear to be resistant to disintegration of the shell structure in acid, although imperfections of the sealing have been noticed.

## 1. Introduction

Gastro-resistant formulations are an example of the most common type of modified drug release systems. Gastro-resistant forms of drug administration allow to:

(1) minimize adverse effects such as nausea and bleeding associated with irritation of gastric mucosa that may be caused by some active substances;
(2) deliver drug intended for local action in intestines;
(3) protect the drug substance from degradation in an acidic environment of the stomach [1].

Gastro-resistant soft gelatin capsules can prove their usefulness in oral administration of drugs of irritating or acid-labile nature, often displaying at the same time enhanced bioavailability in a liquid form, which can be considered an advantage to coated tablets [2]. The most obvious examples of the substances that need to be formulated in gastro-resistant dosage forms are non-steroidal anti-inflammatory drugs (NSAIDs), which are irritating to gastric mucosa.

The products in the form of gastro-resistant capsules usually are designed as conventional hard capsule shells filled with the enteric-coated pellets or minitablets. Manufacturing of gastro-resistant soft capsules, however, is a challenge. Due to the liquid fill, modification of the drug release rate from soft capsules can be achieved only by modification of the capsule shell to make it resistant to acidic pH. This issue can be approached by-coating of standard capsules with acid-resistant polymers such as methacrylic acid—methyl acrylate copolymers (e.g., Eudragit L or S^®^) [3]. A less popular alternative is incorporation of gastro-resistant polymers in the shell material used to form the capsules [4]. Both approaches are technologically perplexing at some points, although modification of the shell material can be considered more beneficial from both economic and technological point of view. However, it is not yet utilized in commercial products. It is substantial to take into consideration that any changes in the composition of the film-forming mixture can result in significant alteration of the overall physicochemical properties of the prepared films, that can lead to the loss of their potential to be formed into capsules in a conventional manufacturing process.

A very important issue associated with the development of a new capsule shell composition is to identify the physiochemical phenomena that can be utilized in designing and manufacturing of modified release gelatin-based films. In our previous work, selection of the most effective modification of the shell material composition was performed, and their microstructure and barrier properties were described [5,6]. However, there are still a few unexplained issues in the description of the phenomena that lead to formation of the films, as well as the changes that the films undergo when exposed to various conditions. Therefore, in the present work, a more detailed investigation of the events associated with the formation of the gastro-resistant film was performed and further, the structural changes upon submersion of such films in acidic dissolution fluid is performed. For the purpose of better characterization of the films and film formation processes, several modern techniques may be employed. In the present research, a scanning electron microscopy (SEM), confocal laser scanning microscopy (CLSM), confocal Raman microscopy and quartz crystal microbalance with dissipation monitoring (QCM-D) were used. Additionally, the barrier properties of the films against oxygen were evaluated.

In comparison to tablets or hard capsules, the transfer of a new technology for soft capsules from the lab to the production site is much more complicated, and a scale-up procedure may be complicated and time-consuming. One of the main issues when soft capsules are developed is a poor access to a lab-scale equipment that could allow to assess the utility of the modified films for capsule formation. The most problematic is the fact that, at a commercial scale, specific rheological and mechanical properties of the film-forming material are required [7,8,9]. The fact that the shell-forming material has to be tested on a large scale, significantly increases the cost of technology development. In our present work, the lab-scale production process of the soft capsules is presented, utilizing a simple mold for suppositories, what allowed to evaluate the shell compatibility with the filling material.

## 2. Materials and Methods

### 2.1. Materials

Components of films and capsules: gelatin type B, bovine hide, 220 bloom (Sigma Aldrich, Saint Louis, MO, USA), glycerol 99.5% w/w (Chempur, Piekary Slaskie, Poland), Aquacoat® CPD (FMC Biopolymer, Philadelphia, PA, USA), aqueous dispersion of cellulose acetate phthalate (CAP), iota-carrageenan (Sigma Aldrich, Saint Louis, MO, USA), medium-chain triglycerides (MCT)—Miglyol 812 N (Caelo, Hilden, Germany), polyethylene glycol 400 (PEG 400)—Kollisolv PEG E400 (Sigma Aldrich, Saint Louis, MO, USA), cetearyl alcohol—TEGO Alkanol 1618 (Evonik, Essen, Germany). Diclofenac sodium was a gift from Polpharma Pharmaceutical Works (Starogard Gdanski, Poland).

Analysis: QCM-D: branched polyethyleneimine Mw 25,000 (Sigma Aldrich, Saint Louis, MO, USA), formaldehyde 37% (Sigma Aldrich, Saint Louis, MO, USA), ethanol 95% (Sigma Aldrich, Saint Louis, MO, USA). Disintegration and dissolution media (0.1 M HCl and phosphate buffer pH 6.8) were prepared according to the European Pharmacopeia 10th edition.

### 2.2. Capsule Formation

**Figure 1:**
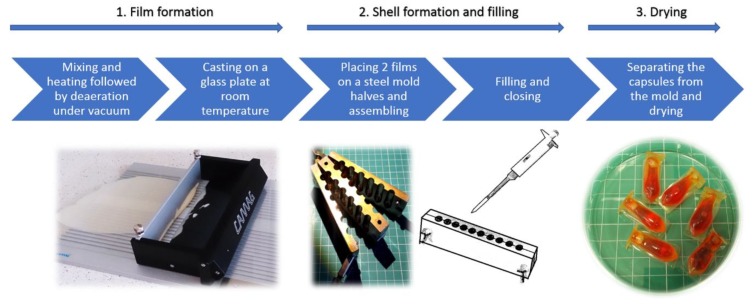
Capsule formation scheme.

Schematic presentation of the capsule formation process is shown in Figure 1.

The preparation method of the film-forming mixtures and films was described in detail in the previously published work [5,6]. Shortly, the mixture of components (Table 1) was stirred at 80 °C for 2 h, which was followed by deaeration under vacuum. Afterwards the mixture was casted on a glass plate using a plate coating device (Camag TLC Plate Coater, Camag, Muttenz, Switzerland) with a height of fluid layer of 1500 μm. After drying the thickness of the film was around 600 µm.

The capsules were prepared using GAC composition (Table 1), by placing 2 pieces of the film (immediately after casting) in a steel form for suppositories. After closing the form, the resulting reservoirs were filled with: (a) MCT oil, (b) PEG 400 or (c) cetearyl alcohol. For a better visual identification of a disintegration test endpoint, the filling material was colored with small amount of a hydrophilic or lipophilic dye. Afterwards, the filling orifice was manually closed with a strip of a film, and to ensure good sealing the capsules were placed for 5 min at 60 °C. Finally, the capsules were stored and dried at ambient temperature and of 15–25% RH for at least 24 h. The measured moisture content in the capsules was around 2.5% (Radwag WPS210S Moisture Analyzer, Radwag, Radom, Poland).

### 2.3. Microscopic Imaging

The imaging of samples was performed with use of a scanning electron microscopy (SEM), confocal laser scanning microscopy (CLSM), confocal Raman microscopy and optical microscopy.

The observation of film samples was performed before and after submersion in 0.1 M HCl at 37 °C, under constant stirring for 2 h (similar to the procedure of swelling test described in our previous work [5]). The films after submersion in acid were frozen in a liquid nitrogen and freeze-dried for 24 h. The investigation was performed with Jeol 7900F SEM (Jeol, Tokyo, Japan), Nikon Ti-E/A1 + CLSM (Nikon, Tokyo, Japan) and WITec Alpha 300 Access Raman microscope equipped with 785 nm laser (WITec, Ulm, Germany).

The imaging of the lab-manufactured capsules was performed using Phenom Pure SEM (Phenom World, Eindhoven, the Netherlands), and Nikon Eclipse 50i optical microscope (Nikon, Tokyo, Japan).

### 2.4. Gas Permeability

The films GEL, GA and GAC (Table 1) were subjected to oxygen permeability tests, performed with an coulometric detector technique according to method ASTM F 1927-14. The equipment used was OX-TRAN 2-20 (Mocon, Minneapolis, MN, USA). The investigated surface was 50 cm^2^.

### 2.5. Quartz Crystal Microbalance with Dissipation Monitoring (QCM-D)

QCM-D was employed to investigate the affinity of CAP latex particles present in Aquacoat CPD to gelatin. The preparation step comprised coating of the gold-plated quartz crystal sensor with branched polyethyleneimine (PEI), then spin-coating the sensor with 1% gelatin solution (5 s at 2500 rpm and low acceleration, followed by 60 s at 8000 rpm and high acceleration). Afterwards the sensor was dried at 60 °C for 20 min. The gelatin on the sensor was subjected to crosslinking by submersion in 1.5% formaldehyde solution, in order to prevent it from dissolving in aqueous conditions. Finally, the sensor was dried at 60 °C for 60 min.

The sensors were mounted in a Qsense equipment (Qsense, Västra Frölunda, Sweden). A deionized water (at 25 °C) was flushed over the sensors until a stable baseline was obtained. Then the diluted (0.1%) Aquacoat CPD at 25 °C was pumped through the cells, and the changes in fundamental frequency overtones of the crystal were registered. After stabilization of the system, the cells were once again pumped with deionized water to remove all the substances that were not bound to the film.

Additionally, to assess the surface structure and stability of the gelatin films obtained in situ on the sensors, the Atomic Force Microscopy (AFM) was performed with NTEGRA Prima setup (NT-MDT Spectrum Instruments, Moscow, Russia), with a silicon probe (spring constant of 40 N m^−1^ and resonant frequency of 300 kHz) (Tap 300AI-G, Budget Sensors, Sofia, Bulgaria). The images were analyzed using a Gwyddion software (Version 2.55, Free Software Foundation, Boston, MA, USA).

### 2.6. Disintegration Time

Disintegration time test of the capsules filled with PEG-400, MCT oil or cetearyl alcohol, was performed. The test was performed using: (a) a tablet disintegration tester ED-2SAPO (Electrolab, Mumbai, India); (b) a paddle dissolution apparatus DT800 (Erweka, Langen, Germany), with a capsule placed in a steel sinker (the stirring rate was 50 rpm). The capsules were tested for 120 min in 0.1 M HCl, followed by pH 6.8 phosphate buffer until disintegration.

### 2.7. Drug Release Test

**Figure 2:**
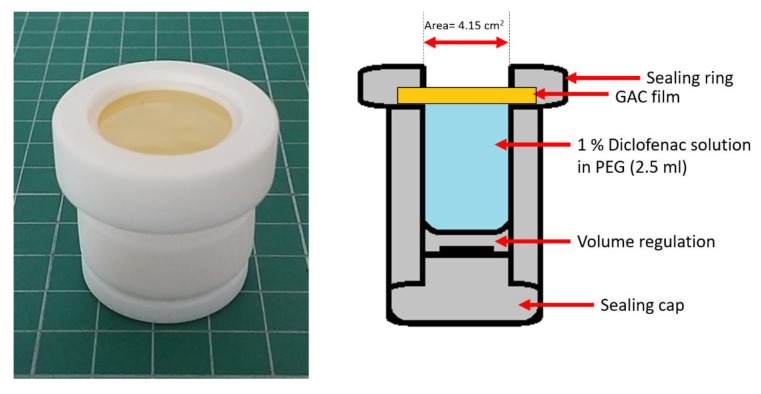
The enhancer cell with gelatin + Aquacoat + carrageenan (GAC) film.

The study was performed using a vertical diffusion cell (Enhancer cell, Erweka, Langen, Germany) and a paddle dissolution apparatus DT800 (Erweka, Langen, Germany) equipped with a built-in autosampler. The stirring rates of 50, 100 and 150 rpm were used. The enhancer cell with the mounted modified gelatin film is shown in Figure 2.

The film selected for the test was gelatin + Aquacoat + carrageenan (GAC), the same as for disintegration tests. The diffusion cell was filled with 2.5 mL of a 1% diclofenac solution in PEG 400 (the amount of diclofenac sodium was 25 mg). Then the investigated film (cut to a circle of 3 cm in diameter) was carefully placed on the top of the solution and secured with a sealing ring and a screw cap; the active surface was 4.15 cm^2^. The test was performed in 900 mL of 0.1 M HCl for 120 min followed by 900 mL phosphate buffer pH 6.8 for 60 min.

Sampling of the acceptor fluid was performed every 15 min in the acid phase, and every 5 min in the buffer phase. Quantification of diclofenac was performed spectrophotometrically at 276 nm wavelength. The study was performed in triplicates.

## 3. Results

### 3.1. Microscopic Imaging of the Films and Capsules

**Figure 3:**
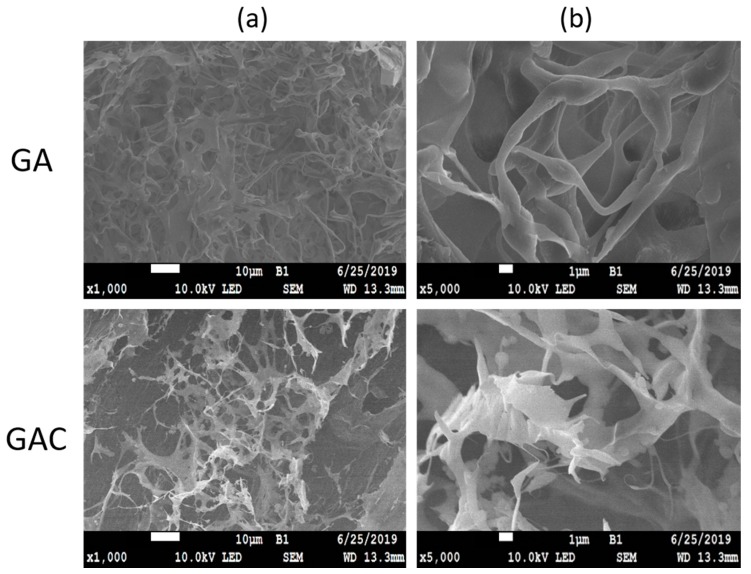
SEM image of GA and GAC films after 2 h in 0.1 M HCl. Scale bar: (**a**) 10 µm, (**b**) 1 µm.

At the first stage of the study, the prepared GA and GAC films were observed prior to and after submersion in HCl. Macroscopically it was visible that the samples after the acid treatment became opaque and swollen. Under the microscope, the untreated samples had a smooth surface with no structures visible [6]. As presented in Figure 3, the films after submersion in acid revealed a network-like structures, resembling scaffolds.

**Figure 4:**
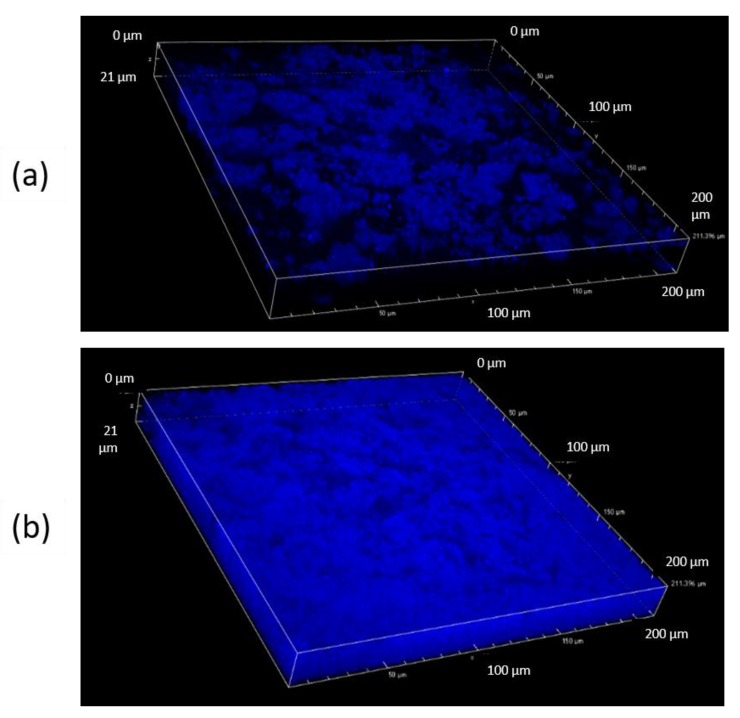
Confocal laser scanning microscopy (CLSM) images of GA film after 2 h immersion in 0.1M HCl: surface layer (**a**) and the inner central part (**b**) of the film.

There are clear differences between the images of a top and a middle layer of the sample (Figure 4). It appears that, after 2 h in acid, noticeably less solid material is left on the top of the film, than in the deeper part. The signals registered by CLSM can be potentially both from CAP and gelatin, due to very similar autofluorescence behavior. However, it is suspected that the outer layer consists mostly of CAP, while in the inner layer a swollen and undissolved gelatin can be present as well.

**Figure 5:**
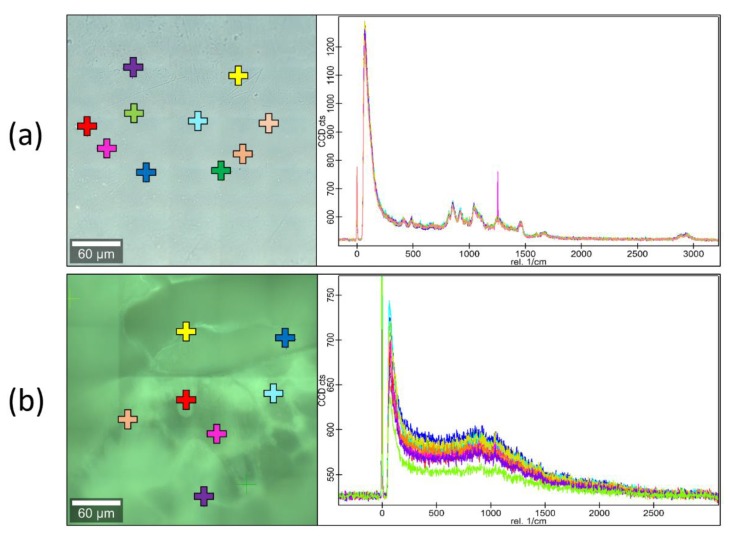
The Raman spectra of several points examined on the surface of GAC film: (**a**) before immersion in acid; (**b**) after immersion for 2 h in 0.1M HCl. Multiple overlaid spectra are presented on each graph.

**Figure 6:**
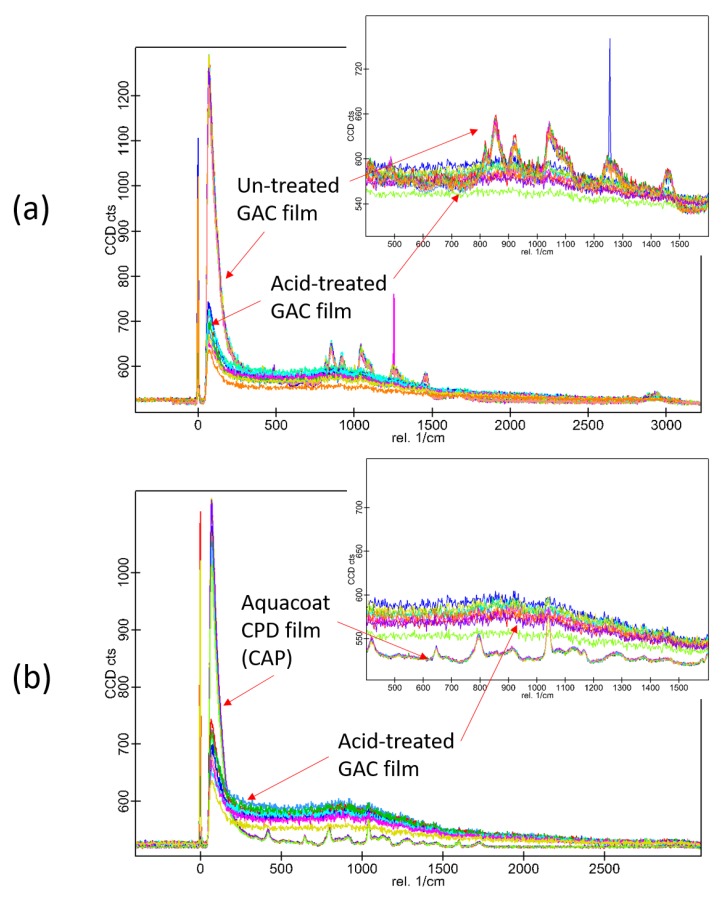
Comparison of the Raman spectra: (**a**) untreated and acid-treated GAC; (**b**) acid-treated GAC and cellulose acetate phthalate (CAP) film (without gelatin). Multiple spectra of each composition are presented.

Raman microscopy investigation was performed on GAC films before and after immersion in 0.1 M HCl. Several points have been scanned to obtain Raman spectra, which have been overlaid and compared. The spectra are shown in Figure 5 and Figure 6.

As it can be seen from the spectra in Figure 5, the surface of the GAC sample is chemically uniform, without any phase separation visible. The acid-treated GAC samples display similar pattern in the spectrum as the untreated GAC. The spectra of the non-modified film (GEL) are not presented in the figure, but they were not different from the spectra of GAC. In Figure 6, different sets of spectra are overlaid. It appears that, in the untreated samples, the gelatin signals are overlapping with the peaks of CAP. After the acid-treatment, the signals from gelatin are weaker, but the signals from CAP are yet undetectable. This outcome can be explained by presence of a small amount of gelatin-rich phase residue undissolved in acid and covering the CAP scaffold. This corresponds well with SEM and CLSM results described above.

### 3.2. QCM-D

**Figure 7:**
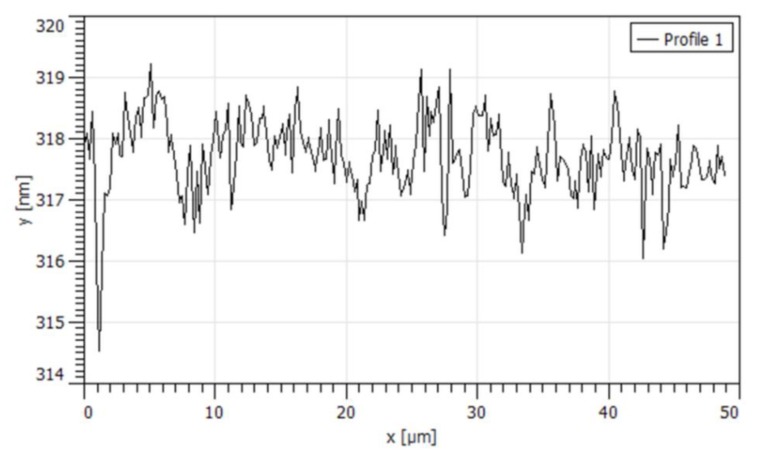
The surface morphology of the gelatin film on the quartz crystal microbalance with dissipation (QCM-D) sensor.

A QCM-D study was performed to obtain supporting information on interactions between gelatin and CAP latex particles. Due to the fact that reliable results regarding particle deposition depend on the morphology of the used substrate, the films obtained in situ on the QCM sensors were investigated with AFM. It was confirmed that the films had uniform thickness and smooth surface, as shown in the Figure 7.

**Figure 8:**
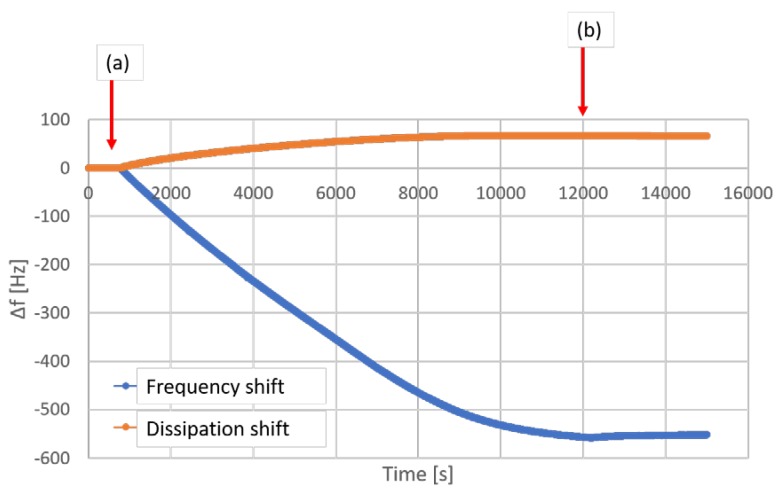
A QCM-D graph obtained at 5th overtone. The approx. 550 Hz drop in frequency carries risk of error on calculating the mass increase: (**a**) start of latex flow; (**b**) start of water flow (to remove particles that are not bound to the film).

The QCM-D graph is shown in Figure 8. Although a large deposition of CAP particles on the gelatin film was detected, the very high extent of the frequency change (around 550 Hz) of all investigated overtones creates a risk of an error when calculating the mass deposition. Therefore, the obtained results were used only for the qualitative, and not for the quantitative analysis.

The deposition of particles proceeded, until the full coverage of the QCM sensor occurred after approximately 12,000 s, which on the graph in Figure 8 is visible as a plateau in the frequency shift. Afterwards, the system was flushed with deionized water for 50 min, what did not cause any significant decrease in the amount of latex particles adsorbed on the gelatin.

### 3.3. Gas Permeability

Oxygen was a gas used for permeability test. Three compositions were investigated (GEL, GA, GAC). The thickness of investigated films was 650 ± 50 µm. The measured oxygen permeability (cm^3^/(m^2^ × 24 h × 0.1 MPa)) was 7.58 for GA sample, 3.43 for GAC and 3.43 for GEL samples. The test was performed twice for all the samples, and the same results were obtained.

### 3.4. Disintegration Time

The results of disintegration time measurements are shown in Table 2. The current pharmacopeial standards (European Pharmacopeia 10th) for disintegration time of gastro-resistant capsules state that the investigated sample should not disintegrate in 0.1 M HCl for 2 or 3 h (depending on the composition, however not less than 1 h), which should be followed by disintegration within 1 h at pH 6.8. In the investigated capsules, at the acid stage, no disruption of the capsule shell material was observed. However, the rupture of the capsule sealing was observed in several capsules.

The results of disintegration time measurements are not significantly different in regard to the method applied. The resistance of capsules to acid was similar when either MCT oil or PEG was used as a filling material. Surprisingly, the capsules filled with cetearyl alcohol disintegrated in acidic conditions within a relatively short time.

A careful observation in acid phase indicated that the shell did not disrupt in any other way but only through the sealing, while the walls of the capsules always retained their integrity. This indicates that the shell material itself is resistant to acid and the filling material does not change this property. The resistance of the capsules to acid, however, lacks reproducibility due to a variability of the seal quality. At pH 6.8, the capsules disintegrated through creation of a breach in the shell, caused by its thinning due to dissolution process. However, similarly to the test in acid, the disintegration always started at the sealing site. It was observed that the disintegration time at pH 6.8 was longer, when the paddle apparatus was used in the test, which can be attributed to different fluid dynamics that had impact on the rate of dissolution of the capsule shell.

**Figure 9:**
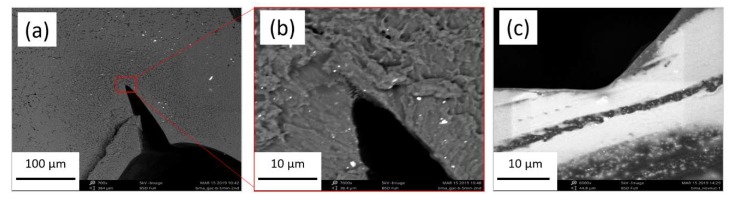
SEM images of the cross-sections of the capsule sealing site: (**a**) an apparently successful sealing; (**b**) close-up of the area; (**c**) a reference commercial soft gelatin capsule.

The sealing sites of the investigated capsule shells and the reference commercial soft capsules were investigated microscopically. Although with an optical microscopy the image of the seals appeared similar to the commercial capsules, in SEM pictures, in some of the prepared capsules more sharp angle at the contact site of the fused films was observed. Such a defect is likely to induce formation of a rupture when capsules are swollen upon submersion in fluid. An evident difference between “commercial” sealing and the lab-scale sealing of the capsules is presented in Figure 9.

### 3.5. Drug Release Test

Due to the fact that during disintegration test, the disruption at the sealing zone appeared as a problem, the drug release test was performed in an enhancer cell, described in the Methods section. Although this system can show potential differences in the kinetics of the drug release in comparison to a filled capsule, still the conclusions about acid-resistance and the rate of drug release can be drawn.

The pharmacopeial standards for drug release test from gastro-resistant forms require the release of less than 10% of the declared drug dose within 2 h in 0.1 M HCl, followed by at least 80% of the dose released at pH 6.8 within a specified time, usually not longer than 45 min.

**Figure 10:**
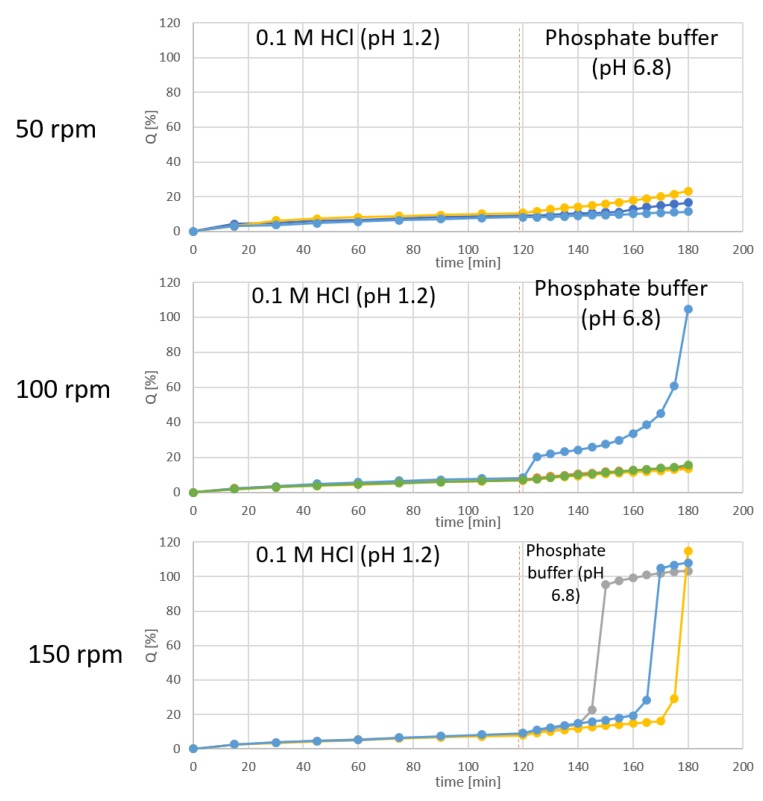
The effect of the stirring rate in a paddle apparatus on the release profiles of diclofenac sodium from the PEG 400 solution in a diffusion cell closed with GAC film.

The drug release test was performed for the diffusion cells filled with 1% m/v solution of diclofenac sodium in PEG (total dose 25 mg of API). The results obtained with different stirring rates are shown in Figure 10.

The results show that at all of the used stirring rates, less than 10% of diclofenac was released during the acid phase of the test, which complies with the pharmacopeial requirements, and confirms the acid-resistance of the investigated films. On the other hand, at the buffer stage of the test, the release occurred in each sample only if they were tested at the high stirring rate, i.e., 150 rpm.

## 4. Discussion

The structures revealed by SEM in the films after they were treated with an acid (Figure 3) allow for conclusion that the formation of the modified films is based on a phase transformation in a gelatin-CAP mixture at a preparation stage. Due to the fact that pH of the utilized type B gelatin solution was around 4.5, one can expect that CAP should constitute a separate phase as this polymer is insoluble at low pH. The CAP phase can be considered continuous; due to the high temperature at the stage of mixture preparation, which is well above the glass transition temperature (Tg) of CAP, the particles can appear in a rubbery state and coagulate easily. Therefore, the two separate phases: gelatin gel and CAP phase, are physically mixed, forming a bi-continuous network with discreet separate microdomains. The structures revealed with SEM suggest that the phase separation proceeds with a spinodal decomposition mechanism, which is spontaneously initiated, and kinetically limited by increase in the viscosity of gelatin during the gelling process when the temperature drops at the casting stage. Similar “kinetic arrestation” of the phase separation process in the gelatin-containing mixtures was described by Lorén et al. [10] and by Tromp et al. [11].

**Figure 11:**
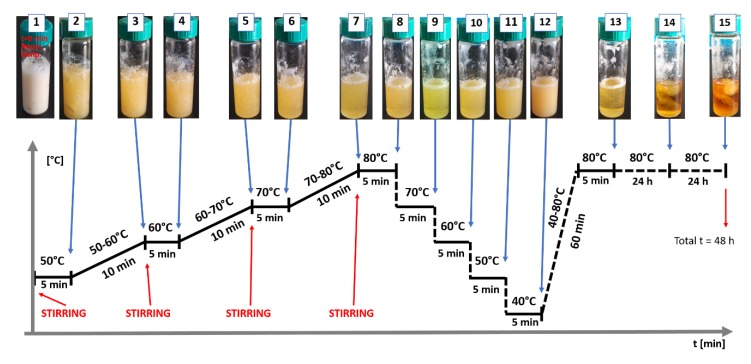
The visible phase-separation on lowering the temperature of the sample, and suspected spinodal decomposition process on storage at 80 °C for a prolonged time.

To better explain the phase separation in the discussed systems, an additional experiment was performed. A premix of GA composition was placed in a glass vial and slowly heated to reach 80 °C. After 5 min at 80°C the temperature was lowered stepwise by 10 °C each 5 min. The appearance of turbidity indicated the phase separation process. After reaching 40 °C the sample was heated again to 80 °C and kept at that temperature for 48 h. The second heating revealed that the temperature-dependent phase separation is reversible (the sample became transparent again). The results are presented in Figure 11.

However, the storage at 80 °C for a prolonged time caused irreversible phase separation, with a fibrillar/sponge-like appearance of the precipitated phase within the liquid continuous phase. Additionally, the alteration of color of the sample and the fact that the gel was partially liquid at room temperature indicated gelatin degradation. The overall results of this experiment confirm that the phase separation process proceeds at high temperature and can be stopped by immobilization of the growing CAP structure in a gelatin gel when the temperature drops below approx. 50 °C. This supports the thesis that the separate phase of CAP acts like a “reinforcement” for the gelatin network, decreasing the rate of penetration of the acidic medium into the water-soluble phase, and explains the structure integrity of the films in acidic media. Furthermore, it appears that the temperature/time balance in the preparation procedure allows to exploit the natural imbalance between phases for the favor of functionality of the films.

The mechanism behind formation and acid-resistance of both binary (GA) and ternary (GAC) polymer systems appears the same. However, in comparison to the GA films, a more significant hindering of disintegration and dissolution can be observed in case of GAC films [5]. This can be explained by a possible interaction between gelatin and carrageenan, which is suspected to be a polyelectrolyte complex formation. This non-covalent interaction has already been reported and is widely described [6,12,13,14,15,16]. Even though carrageenan is present in the film in a small amount, it still can significantly impact the viscosity of the gel phase. Therefore, by increasing the density of the polymer network, it leads to formation of a gel diffusion layer more viscous than the gelatin alone. That causes higher swelling degree and slower dissolution rate of soluble ingredients present in the films, as it was previously described [5]. It appears also possible that the carrageenan in the film-forming mixture accumulates on the CAP-gelatin interface, supporting the CAP scaffold during the immersion in an acid. However, we believe that the more irregular appearance of the CAP scaffolds visible in Figure 4 are actually CAP coated with undissolved gelatin-carrageenan complex. This hypothesis appears to be supported by the results of Raman microscopy (see Figure 6 and Figure 7), in which after immersion in acid, the structure shows a pattern in Raman spectra with the same features as observed in the spectra of the films before the acid treatment (both CAP and GAC), what suggests that during the immersion the gelatin was not fully dissolved and it is still present on the surface of the residual CAP scaffold structure.

The CLSM study (Figure 4) appears to correspond well with the SEM results. Additionally, it was discovered that there are clear differences in the density of the solid material left in structure inside the films and on its surface. Due to the fact that the soluble fraction of the film composition is supposedly gelatin-based gel and plasticizer, the mechanism of erosion of the film when placed in an acid is likely based on the diffusion of the medium through the gel layer. The erosion can be additionally limited by the presence of the insoluble CAP phase, which acts also as a scaffold. Therefore, it can be suspected that the penetration of the acidic medium into the membrane is delayed and can depend on both density of the CAP scaffold and viscosity of the gelatin-based phase.

The higher barrier properties of a ternary system (GAC) than the binary one (GA) also was demonstrated by the oxygen permeability test. However, in the literature the results of oxygen permeability can be found only for very thin gelatin films obtained from dilute gelatin solutions [17], where the values of oxygen permeability can be around 350–600 cm^3^/(m^2^ × 24 h × 0.1 MPa). In the present study the permeability was measured for films with thickness around 650 µm, at which the measured values were between 3.5 and 7.5 cm^3^/(m^2^ × 24 h × 0.1 MPa). Although, after addition of CAP, the oxygen permeability increased slightly, the differences between formulations were still very low and one can conclude that there is a lack of significant influence of the film ingredients on gas barrier properties.

During the formation of modified GA or GAC films, the CAP spherical particles (average size of 0.43 µm) are being incorporated in the gel structure. QCM-D is a surface sensitive technique which can be applied to analyze the interaction of the particles of CAP with gelatin. Quantitative values can be obtained with well-defined model systems. Saurebrey and Johannsman models [18] are often used to calculate the surface excess after adsorption. In our particular case, those models will not give reliable approximation because of the large size of the CAP particles. However, a qualitative information on interaction between CAP particles and gelatin films can be obtained. The large decrease in frequency of the vibration as soon as the CAP particles were introduced into the flow cell reflects the adsorption of CAP particles on the gelatin films. Due to the fact that the measurements were performed at 25 °C, a coalescence of the CAP is rather negligible, therefore such interaction should be based purely on surface charge of the particles. Although the test could not be performed at high temperature (80 °C), the confirmed high affinity of these two materials at 25 °C may be also relevant at higher temperature. We assume that such type of interaction can potentially stabilize the CAP inside the gel matrix and allow formation of a network structure during the preparation of the film-forming mass.

Preparation of capsules on a lab scale with the proposed steel mold was a simple process, allowing for application of a liquid fill, and for obtaining visually sealed capsules. However, the results of the disintegration tests show large variability because of the significant tendency of capsules to disrupt at the sealing area. On the other hand, the results prove that formation of the capsules using GAC composition is generally possible, and the capsules can be filled with liquid oil, PEG or melted fatty alcohol. In addition, no case in which a capsule disintegrated in acid at other region than the sealing was observed. This confirms that the films being in contact with a filling, still retain their structural integrity when submersed in acid.

For the purpose of investigation, whether the filling formulation has an impact on acid-resistance of the capsule shell, the capsules were filled with three types of substances: PEG, MCT oil and cetearyl alcohol. The results show that the capsules filled with solid fatty alcohol show lower resistance of the sealing to disintegration in acid. On the other hand, there is no noticeable difference between the capsules filled with MCT oil or PEG. Overall, the mechanism of disintegration of the capsules appears to be related more to the capsule formation process, than to the filling composition. It was observed that the lab-manufactured capsules are prone to leakages on the sealing zone, especially when more intensive mechanical stress was involved, as in the tablet disintegration apparatus. We believe that the imperfect capsule sealing can be corrected when encapsulation process involves the conventional soft capsule manufacturing machines.

The imperfections in the sealing region did not allow to further test the capsules in the drug release test. This is why this study was performed in a vertical diffusion cell placed in a paddle dissolution apparatus. The test was performed to investigate whether the films display barrier properties against diffusion of diclofenac sodium at acidic pH. In addition, it was important that the films allow to release the API after switching the pH to neutral (6.8). In one of our previous articles, the barrier properties of the films towards radio-labeled water were described [5], and preliminary data on the diffusion-hindering by the modified gelatin-CAP compositions was obtained. The present investigation confirms appropriate barrier properties of GAC film, because no diffusion of diclofenac during 2 h in 0.1 M HCl was observed. However, the reproducibility of the diclofenac release at pH 6.8 is not very high. The release was initiated at different time points, what results from the mechanism of film rupture—not dissolving totally in a specified time, but forming a breach. Since at lower stirring rates the release of diclofenac did not occur or was accidental, the proposed model requires higher stirring rates, which shows the significance of the mechanical factor in the dissolution of the GAC film in the pH 6.8 buffer.

Although the performed experiment with diclofenac as a model drug demonstrates lack of the drug diffusion through the modified gelatin film immersed for 2 h in an acid, diffusion of an acid through the membrane was not measured in the course of this stage of the research. Impermeability of the new capsule-forming material to the acid is a condition for using it in the capsules filled with an acid-labile drugs.

## 5. Conclusions

In this work the discreet kinetically-limited phase separation was identified as the main factor influencing the resistance of the modified gelatin films to disintegration in the acidic environment. The imaging techniques (SEM, CLSM, Raman microscopy) provided the information on the mechanism of film partial dissolution in acid. The QCM-D analysis proved the affinity of the latex particles to the surface-wetted gelatin structures, which may be important in regard to the film formation process. The lab-scale soft capsule formation process was performed, and the tested filling materials were proved to be compatible with the films, however the obtained capsules showed the sealing area as a weak spot, limiting the acid-resistance of the capsules during the disintegration test. On the other hand, a modified dissolution test with a paddle apparatus and diffusion cell allowed to confirm that the films are hampering the drug release in acidic phase, while releasing the drug at pH 6.8. The drug release at pH 6.8 was possible, however, only when higher stirring rates (150 rpm) were applied.

## Figures and Tables

**Table 1:** Compositions of the films.

Composition Symbol	Mixture Components (g/100 g)				
	Gelatin	Aquacoat CPD	Iota-Carrageenan	Water	Glycerol
GEL ^1^	41.2	−	−	40.0	18.8
GA ^2^	30.9	34.4	−	15.9	18.8
GAC ^2^	30.0	34.4	0.9	15.9	18.8

^1^ non-modified film (reference); ^2^ modified binary (GA); and ternary (GAC) polymer films.

**Table 2:** Disintegration time of GAC capsules with various fill. Three capsules from each batch were subjected to the test. The results are shown as mean ± standard deviation.

The GAC Capsule Filling Type	Batch No.	Tablet Disintegration Tester		Paddle Apparatus	
		0.1 M HCl	Phosphate Buffer pH 6.8	0.1 M HCl	Phosphate Buffer pH 6.8
MCT oil	1	86 ± 7 min	n/a	>2 h	6 ± 4.6 min
	2	>2 h	3.7 ± 1.5 min	>2 h	10.7 ± 7.4 min
	3	>2 h	2.5 ± 0.7 min	Single capsule leaking ^1^	6.5 ± 7.8 min
PEG 400	1	77.7 ± 7.0 min	n/a	Single capsule leaking ^1^	12.7 ± 4.2 min
	2	>2 h	7.3 ± 1.5 min	>2 h	39.0 ± 18.5 min
	3	>2 h	4.0 ± 2.0 min	>2 h	43.0 ± 13.2 min
Cetearyl alcohol	1	45.7 ± 7.4 min	n/a	79.3 ± 16.3 min	n/a
	2	48.7 ± 16.0 min	n/a	82.7 ± 23.3 min	n/a
	3	29.0 ± 33.0 min	n/a	81.3 ± 18.5 min	n/a

^1^ the leakage was observed on the sealing of capsule.
